# Effects of varying sources of Cu, Zn, and Mn on mineral status and preferential intake of salt-based supplements by beef cows and calves and rainfall-induced metal loss

**DOI:** 10.1093/tas/txab046

**Published:** 2021-03-07

**Authors:** John D Arthington, Maria L Silveira, Luana S Caramalac, Henrique J Fernandes, Jeff S Heldt, Juliana Ranches

**Affiliations:** 1 Department of Animal Sciences, University of Florida, Gainesville, FL 32611, USA; 2 Range Cattle Research and Education Center, University of Florida, Ona, FL 33865, USA; 3 State University of Mato Grosso do Sul, Aquidauana, MS 79804–970, Brazil; 4 Micronutrients USA, LLC, Indianapolis, IN 46231, USA

**Keywords:** calves, supplementation, trace minerals

## Abstract

Three studies were completed to evaluate the effects of Cu, Zn, and Mn source on preferential intake, trace mineral status, and rainfall-induced metal loss of salt-based mineral supplements. Mineral supplements were formulated to contain 2,500, 5,500, and 4,000 mg/kg of Cu, Zn, and Mn, respectively. Supplements differed only by source of Cu, Zn, and Mn, which were hydroxychloride, organic, or sulfate sources. In Exp. 1, the three formulations were offered simultaneously for 18 wk to preweaned beef calves (four pastures; 17 calves per pasture) within separate containers inside covered cow-exclusion areas. Consumption averaged 21 ± 2.4 g/calf daily (sum of all three sources), with a greater (*P* < 0.001) percentage of the total intake coming from the hydroxychloride vs. organic or sulfate sources of Cu, Zn, and Mn. In Exp. 2, the same sulfate and hydroxychloride formulations were randomly assigned to pastures (*n* = 4 pastures per treatment) containing 18 to 20 cow–calf pairs/pasture. Treatments were offered for 20 wk within covered areas designed to assess cow and calf intake separately. At weaning, liver biopsies were collected from four cow–calf pairs/pasture (*n* = 16 cows and calves per treatment). Source of Cu, Zn, and Mn had no effect on voluntary mineral intake among calves (*P =* 0.44) and cows (*P =* 0.14). Calves consuming mineral containing hydroxychloride sources of Cu, Zn, and Mn tended (*P =* 0.06) to have greater average daily gain over the 20-wk period compared with calves consuming sulfate sources of the same elements (1.09 vs. 1.06 kg/d; SEM = 0.013). Mineral status of cows and calves was not affected (*P* ≥ 0.17) by source of Cu, Zn, and Mn. In Exp. 3, each of the mineral formulations from Exp. 1 was exposed to a 10.2-cm precipitation event delivered in three equal 3.4-cm applications within a week. To accomplish this, 750 g of mineral was placed into Buchner funnels (177 cm^2^) on 20- to 25-µm pore filter paper. Deionized water (pH adjusted to 5.6) was poured over the mineral. Total leaching losses of Cu, Zn, and Mn were less (*P* < 0.001) for formulations containing hydroxychloride vs. organic and sulfate sources. These results imply that, when offered a choice, calves preferentially consume mineral supplements formulated with hydroxychloride vs. sulfate or organic sources of Cu, Zn, and Mn. In addition, hydroxychloride sources of Cu, Zn, and Mn are less susceptible to rainfall-induced leaching losses compared with sulfate and organic sources.

## INTRODUCTION

Cow/calf production systems are typically pasture- or range-based relying on forage to supply the majority of nutrient requirements. In some cases, forage alone is inadequate to support minimum nutrient requirements resulting in the need for supplementation (Kunkle et al., 2000). Most notable in grazing cow/calf production systems is the practice of providing free-choice, salt-based mineral supplements. This practice is based on the concept that cattle will seek and consume salt at amounts that meet and exceed their Na requirement (Morris, 1980). As a result of this free-choice consumption behavior, ingredients that deliver other limiting minerals are blended into salt-based mixtures and supplemented to cattle. Applications, strategies, and limitations of free-choice mineral supplementation of grazing cattle have been previously reviewed (McDowell, 1996; Greene, 2000).

In most production systems, preweaned calves have access to the same free-choice mineral supplements consumed by their dams; however, intake is not well understood. In a previous study, we reported a reluctance for voluntary intake of mineral-fortified creep feed among preweaned calves (Moriel and Arthington, 2013). The authors speculated that intake aversion was related to the mineral fortification of these supplements because Control supplements, without mineral inclusion, were readily consumed. However, the authors also indicated that mineral-fortified supplement not consumed in the study was offered and readily consumed by a group of adult beef cows not enrolled in the experiment. This dichotomy of intake behavior led to the development of our hypothesis that young calves possess acute taste sensitivity that is lessened with advancing age. Indeed, cattle are known to have a very sensitive sense of taste (Davies et al., 1979) compared with humans and other domestic animals (Roura et al., 2008), which may decrease with advancing age (Boyce and Shone, 2006). In a series of follow-up experiments, we evaluated preferential intake of mineral-concentrated supplements containing differing sources of Cu, Zn, and Mn. In those studies, calves displayed a large preference for consumption of mineral-concentrated feeds containing lesser soluble sources of Cu, Zn, and Mn (i.e., hydroxychloride sources) vs. more soluble sources (i.e., organic or sulfate) of the same elements (Caramalac et al., 2017). The objectives of the current studies were to investigate intake and mineral status of beef cows and calves and rainfall-induced metal leaching loss of free-choice, salt-based, mineral supplements formulated with hydroxychloride, organic, or sulfate sources of Cu, Zn, and Mn.

## MATERIALS AND METHODS

Three experiments were designed to investigate measures of intake, mineral status, preweaning performance, and metal leaching losses of salt-based supplements containing differing sources of Cu, Zn, and Mn. The experiments were conducted at the Range Cattle Research and Education Center at Ona, FL. Animal care and handling for Exp. 1 was performed according to *The Guide for the Care and Use of Agricultural Animals in Research and Teaching* (FASS, 2010). Procedures for Exp. 2 were reviewed and approved by the University of Florida, Institutional Animal Care and Use Committee (IACUC No. 201508930). There were no animal subjects used in Exp. 3.

### Animals, Diets and Management

Experiments were conducted using commercially manufactured, free-choice, salt-based mineral supplements (Provimi North America, Brookville, OH). The supplements were formulated to be nutritionally identical. Inclusion level of Ca carbonate was adjusted to account for differences in metal content among the three sources of Cu, Zn, and Mn (Tables 1 and 2).

**Table 1.** Ingredient composition of limit-fed supplements (Exp. 1 and 2)^1^

Item	Organic^2^	Hydroxychloride^3^	Sulfate^4^
	% (as-fed basis)		
Ca carbonate	33.01	39.23	37.84
NaCl	23.00	23.00	23.00
Monocalcium phosphate^5^	18.82	18.82	18.82
Mg mica^6^	12.36	12.36	12.36
Dried molasses	2.50	2.50	2.50
Mineral oil	1.00	1.00	1.00
Hydroxychloride Zn	0	0.91	0
Zn sulfate	0	0	1.41
Organic Zn	3.33	0	0
Hydroxychloride Mn	0	0.91	0
Mn sulfate	0	0	1.27
Organic Mn	2.67	0	0
Hydroxychloride Cu	0	0.46	0
Cu sulfate	0	0	0.99
Organic Cu	2.50	0	0
Vitamin E (50%)	0.09	0.09	0.09
Ca iodate	0.06	0.06	0.06
Vitamin A (1,000,000 U)	0.04	0.04	0.04
Na selenite	0.60	0.60	0.60
Co carbonate	0.01	0.01	0.01
Vitamin D3 (500 kIU/g)	0.01	0.01	0.01
Total	100	100	100

^1^Supplements formulated to be as nearly identical as possible. Calcium carbonate inclusion was altered to account for differences in metal concentration of ingredients supplying Cu, Zn, and Mn. Targeted specifications = 2,500, 5,000, 4,000, 60, and 60 mg/kg for Cu, Zn, Mn, Co, and Se, respectively.

^2^Organic ingredients = 15.0%, 15.0%, and 10.0% Zn, Mn, and Cu, respectively. Organic formulation was used in Exp. 1 only.

^3^Hydroxychloride ingredients = 55.0%, 44.0%, and 54.0% Zn, Mn, and Cu, respectively.

^4^Sulfate ingredients = 35.5%, 31.0%, and 25.2% Zn, Mn, and Cu, respectively.

^5^BioFos (21% P; Mosaic Feed Ingredients, Lithia, FL).

^6^Micro-Lite (MicroLite LLC., Buffalo, KS).

**Table 2.** Chemical composition of salt-based, mineral supplements (dry matter basis)^1^

	Exp. 1			Exp. 2	
Item	Hydroxychloride	Sulfate	Organic	Hydroxychloride	Sulfate
Ca, %	18.47	15.81	16.26	19.30	19.33
P, %	3.57	5.04	4.11	3.80	3.38
Mg, %	1.37	1.35	1.15	1.23	1.15
K, %	0.43	0.52	0.55	0.39	0.42
Na, %	8.50	8.33	9.18	8.47	7.48
S, %	0.42	1.00	1.00	0.47	1.28
Fe, mg/kg	7,543	9,200	7,717	7,680	7,520
Zn, mg/kg	6,205	5,180	4,957	8,380	9,030
Cu, mg/kg	2,458	2,287	2,423	3,410	3,290
Mn, mg/kg	4,265	3,733	3,787	5,500	6,440
Mo, mg/kg	2.20	2.77	2.30	2.70	2.20
Co, mg/kg	68.2	75.0	57.9	132.9	161.3
Se, mg/kg	57.0	56.3	55.6	110.4	176.8
DCAD	−29	−58	−50	−54	−96

^1^Results are a mean of 3 and 6 individual analyses, Exp. 1 and 2, respectively, derived from random hand-grab samples.

**Figure 1.**
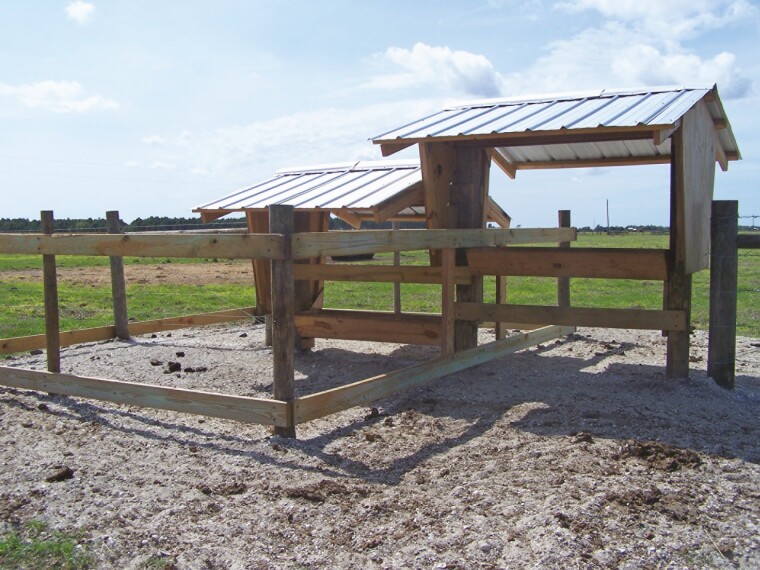
Mineral feeders used in Exp. 1 and 2 were designed to assess mineral intake of cows and calves independent of each other. Calves are excluded by height of the mineral feeder (approximately 100 cm), and cows are excluded by a creep gate restricting entry (approximately 75 cm height). Both feeders are covered by a slanted roof approximately 120 cm above the mineral feeder with the roof edge extending approximately 100 cm from the edge of the mineral feeder.

Experiments 1 and 2 were conducted using the same pasture groups and cowherds in consecutive years. The cowherd consisted of Brangus-crossbred (approximately 20% Brahman) pregnant to Brangus and Angus bulls. Pastures consisted of established bahiagrass (*Paspalum notatum*), which were sampled in both years in April, May, and June. In each of these months, random, hand-plucked samples were collected from each pasture, pooled into a single sample, and sent in duplicate to a commercial laboratory for chemical composition analysis (Dairy One Laboratory, Ithaca, NY). Calves are born over an approximate 90-d interval beginning in early October. The mineral feeders in these pastures were constructed to assess cow and calf mineral intake individually. For cows, calves were excluded by height of the mineral feeder, and for calves, cows were excluded by a creep gate, which excluded cow entry. Mineral feeder specifications is provided in Figure 1.

The objective of Exp. 1 was to evaluate differences in preferential intake among preweaned beef calves provided free-choice, salt-based, mineral supplements formulated with hydroxy, organic, or sulfate sources of Cu, Zn, and Mn. The three formulations were offered simultaneously to preweaned beef calves (four pastures; 17 calves per pasture) within separate stainless steel bowls inside covered cow-exclusion areas within each of the study pastures; therefore, all pastures received the same access to each treatment formulation to allow measurement of preferential intake. Beginning 7 March, preferential intake was evaluated weekly for 18 wk prior to weaning. Calves were approximately 100 d old at the start of the study. Fresh supplements, in amounts to ensure free-choice intake, were provided weekly. Unconsumed supplements were collected, weighed, sampled for dry matter (**DM**) and discarded.

The objective of Exp. 2 was to evaluate intake and mineral status among cows and preweaned calves provided free-choice, salt-based mineral supplement containing either hydroxy or sulfate sources of Cu, Zn, and Mn. The two mineral treatments were randomly assigned to pastures (*n* = 4 pastures per treatment) containing 18 to 20 cow–calf pairs/pasture. Treatments were delivered to pastures within covered cow and calf exclusion areas, which were designed to allow intake measures separately. Calves were born over a 90-d period (October–December) and were an average age of 96 d and 126 kg when the study began (9 February). Voluntary intake was evaluated weekly for 20 wk prior to weaning. Fresh supplement, in amounts to ensure free-choice intake, was provided weekly. Unconsumed supplement was collected, weighed, sampled for DM, and discarded. Intake (g/d) was calculated by the difference between DM of mineral offered and DM of mineral refused divided by the number of cows and calves per pasture. Calves were held off pasture in a drylot pen for approximately 16 h prior to measuring body weight (**BW**). Calves were not separated from cows during this time. Calf average daily gain (**ADG**) over the 20 wk-period was adjusted for sex by calculating the difference in the average BW of heifers and steers and then divided by two. This value was added (if the gender group was lesser) or subtracted (if the gender group was greater) from the individual calf BW. To estimate mineral status liver biopsy samples were collected from 4 random cow and heifer calf pairs in each pasture (*n* = 16 cows and calves/treatment) at the end of the study. Liver tissue was collected by a trained technician using techniques previously described (Arthington and Corah, 1995). Samples were collected between the 11th and 12th intercostal space using a Tru-Cut biopsy needle (CareFusion, 14 gauge × 15 cm; Becton Dickinson, Vernon Hills, IL). Three to four core tissue samples were collected from each animal. Following collection, samples were frozen at −20 °C and sent to the Animal Health Diagnostic Laboratory, Michigan State University (Lansing, MI) for analysis of trace mineral concentration using inductively coupled plasma-atomic emission spectroscopy techniques as described by Braselton et al. (1997).

Experiment 3 was conducted to estimate the amount of Cu, Zn, and Mn lost due to rainfall-induced leaching. The test material was obtained from three random 22.7-kg bags collected from the same manufacturing runs used to create the mineral supplements for Exp. 1 (Table 1), with chemical composition determined separately (Table 3). These experimental formulations differed only by the source of Cu, Zn, and Mn included in the formula, which were hydroxychloride, organic, or sulfate sources. Rainfall simulations were conducted by exposing each mineral formulation to a 10.2-cm precipitation event delivered in three equal applications within a week (3.4 cm on Monday, Wednesday and Friday). To accomplish this, 750 g of mineral was placed into Buchner funnels (177 cm^2^) on 20- to 25-µm pore filter paper (four replicates per treatment). Deionized water (pH adjusted to 5.6) was poured over the mineral and the leachate collected into volumetric flasks and analyzed for Cu, Zn, and Mn concentration using inductively coupled plasma spectroscopy technique (Dairy One, Ithaca, NY). Metal loss was estimated by subtracting the mass of Cu, Zn, and Mn from the original product and the final leached material. Results are expressed as lsmeans (cumulative metal loss; % of initial) for each of the three leaching events.

**Table 3.** Assayed concentration of Zn, Cu, and Mn prior to leaching (mg/kg DM; ± SD; Exp. 3)^1^

Form	Zn	Cu	Mn
Hydroxychloride	6,530 ± 569.3	2,630 ± 260.6	4,423 ± 480.0
Sulfate	5,400 ± 36.1	2,440 ± 10.0	4,067 ± 107.9
Organic	5,130 ± 190.8	2,567 ± 45.1	3,960 ± 147.3

^1^Average ± SD of triplicate analyses. The material used in Exp. 3 was obtained from a single 27.7-kg bag from the manufactured run performed for Exp. 1. The formulation specifications are provided in Table 1.

### Statistical Analyses

Data were analyzed as a completely randomized design using the MIXED procedure of SAS (SAS Institute Inc., Cary, NC) and Satterthwaite approximation to determine the denominator degrees of freedom for the tests of fixed effects. Analysis of supplement intake (Exp. 1 and 2) was achieved using repeated measures with treatment, week, and treatment × week in the model statement. Pasture was the experimental unit and pasture(treatment × week) as the random effect. Covariance structure was determined using the lowest Akaike information criterion. Treatment mean comparisons were achieved using PDIFF. For measures of calf ADG and cow and calf liver trace mineral concentrations, the model statement included treatment and pasture(treatment) as the random effect. Calf ADG was corrected for outliers by removal of data points > 2× the SD of the mean for each treatment (*n* = 2 and 4 for hydroxy and sulfate treatments, respectively). Calf BW at the start of the study was used as a covariate to assess overall BW gain. For all variables analyzed, pasture was the experimental unit. For Exp. 3, analysis of metal leaching loss was assessed using repeated measures with mineral source, leaching event, and mineral source × leaching event in the model statement. Each experimental apparatus was considered an experimental unit and apparatus (mineral source) as the random effect. For each experiment, significance was set at *P* ≤ 0.05, and tendencies if *P* > 0.05 and ≤ 0.10. Results are reported according to main effects when interactions were not significant.

## RESULTS AND DISCUSSION

Ingredient formulation and chemical composition of the free-choice, salt-based mineral supplements used in these experiments are provided in Tables 1 and 2, respectively. Chemical composition of the pasture forage is provided in Table 4. A single manufacturing run was made for each experiment. In Exp. 1, the chemical composition of the final product was similar to the formulation specifications with the exception of Zn, which was 18% greater for the product containing Zn hydroxychloride than the average of the products containing Zn sulfate and organic Zn. In Exp. 2, concentrations of Cu, Zn, Mn, Co, and Se were greater than the targeted formulation specification (average = 34%, 74%, 49%, 145%, and 140% greater amounts of Cu, Zn, Mn, Co, and Se in the final products vs. formulation specification). Although greater than targeted specification, assayed concentrations of the elements of specific interest (Cu, Zn, and Mn) differed only slightly between the hydroxychloride and sulfate treatments (3.5%, 7.2%, and 14.6% for Cu, Zn, and Mn, respectively). These products did differ more substantially for final concentrations of Co and Se, which were 17.4% and 37.9% greater in the sulfate vs. hydroxy treatment (Table 2).

**Table 4.** Chemical composition of pasture forage (Exp. 2; dry matter basis)^1^

Nutrient^2^	Exp. 1	Exp. 2
CP, %	17.0	18.3
ADF, %	32.9	33.1
NDF, %	66.2	66.0
Ca, %	0.39	0.40
P, %	0.31	0.35
Mg, %	0.29	0.25
K, %	1.87	2.01
Na, %	0.009	0.009
S, %	0.33	0.32
Fe, mg/kg	106	98
Zn, mg/kg	31	45
Cu, mg/kg	11	11
Mn, mg/kg	39	57
Mo, mg/kg	0.60	0.57

^1^Results are an average of three samples collected in April, May, and June.

^2^CP = crude protein; ADF = acid detergent fiber; NDF = neutral detergent fiber.

**Figure 2.**
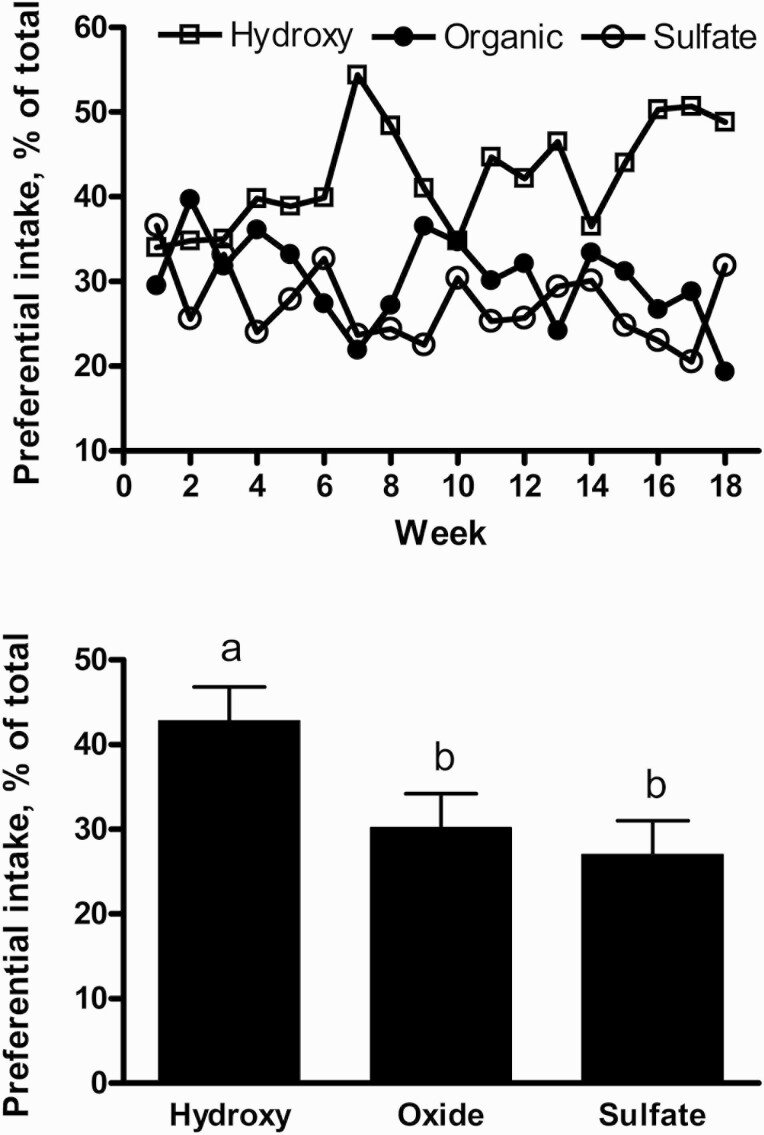
Preferential intake of three salt-based, free-choice mineral supplements offered to calves simultaneously over an 18-wk period. Means sum to 100%. Treatment × time (*P <* 0.001; Pooled SEM = 6.21) response illustrated by top figure. This response appeared to be affected by heavy rainfall events, whereas preferential consumption of the hydroxychloride treatment was reduced in the weeks when the mineral offer was rain soaked (observation only; no data collected). Supplements were formulated to differ only by source of Cu, Zn, and Mn. Pooled means with different letters (a,b) differ (*P <* 0.05; bottom figure).

In Exp. 1, there was a treatment × week interaction (*P <* 0.001) for preferential intake of free-choice mineral (Figure 2). This response was likely affected by excessive rainfall events, whereas preferential consumption of the hydroxychloride treatment was reduced in the weeks when the mineral offer was rain soaked (observation only; data not collected). Although the mineral feeders were covered and provided some protection from the weather, the roofline both above and over the feeder was often insufficient to protect against blowing rain (Figure 1). Consumption of mineral averaged 21 ± 2.4 g/d (sum of all 3 sources). Averaged over the 18-wk experimental period, greater (*P* < 0.001) total intake was associated with formulation containing hydroxychloride vs. organic or sulfate sources of Cu, Zn, and Mn (42.8%, 30.2%, and 27.0% of total intake, respectively; SEM = 1.03; Figure 2). This response is similar to our previous study investigating mineral-fortified, limit-creep supplements for calves (Caramalac et al., 2017). In that study, young calves preferentially consumed creep feed supplements containing concentrated amounts of Cu, Zn, and Mn from hydroxychloride sources vs. sulfate or organic sources. Corroborating our results, previous studies reported weaned pigs also displayed preferential intake of diets formulated with hydroxychloride vs. sulfate sources of Cu, but the preferential response was not as pronounced as the calf studies and decreased with increasing days of exposure to experimental diets (Van Kuijk et al., 2019; Villagomez-Estrada et al., 2020). We hypothesized that the response observed in the current study was due to lesser solubility of the hydroxychloride forms of these metals, particularly compared with sulfate and organic alternatives. Copper sulfate is almost fully soluble in water, whereas Cu hydroxychloride is virtually insoluble (Spears et al., 2004). Thus, the greater solubility allows for greater dissolution in the mouth potentially resulting in an adverse taste reaction. Oronasal nutrient sensing is largely based on both smell and taste sensory mechanisms (Roura et al., 2008). Although previous comparative biology studies have shown that cows have nearly 3× the number of olfactory receptor genes compared with humans, nearly 1/2 of these have become nonfunctional during the period of mammalian evolution (Niimura and Nei, 2007). These changes have very likely resulted in the bovine’s adaptation toward taste sensory as the primary nutrient sensing mechanism. This sensory mechanism is well developed with over twice the number of taste buds compared with humans and over 11× the number of taste buds compared to dogs (Roura et al., 2008). Although the taste sensory to differing metal sources was not specifically studied in the current experiments, these reports support the theory of a “metallic-taste” sensitivity (Epke et al., 2009) in calves consuming mineral-fortified supplements containing soluble sources of Cu, Zn, and Mn.

When provided only a single treatment and cattle had no option for preferential selection (Exp. 2), there were no treatment × week (*P =* 0.84) or treatment (*P =* 0.14) effects for voluntary free-choice mineral intake among cows or calves (average = 16.1 and 65.1 g/d for calves and cows, respectively; SEM = 1.31 and 3.64; Table 5). Cows and calves voluntarily consumed mineral at an approximate rate of 130 mg/kg BW, which is a typical free-choice intake for this region when a 23% salt mineral is offered during the summer months. When offered a single source of mineral, calves likely consumed amounts sufficient to satisfy their salt craving, irrespective of the source of Cu, Zn, and Mn in the formulation. Cattle have a strong appetite for salt and when provided free-choice can be expected to consume salt at a level that meets or exceeds their Na requirement (Berger, 2006).

**Table 5.** Free-choice, salt-based mineral intake among cows and calves (Exp. 2)^1^

	Treatment			
Group	Hydroxychloride	Sulfate	SEM	*P*-value
	g/d			
Cows	69.5	60.7	3.64	0.14
Calves	15.0	16.6	1.31	0.44

^1^Values are lsmeans. Intake measured over 20 consecutive weeks from eight pastures (*n* = 4 pastures per treatment) containing 18 to 20 cow–calf pairs/pasture.

Calves consuming mineral containing hydroxychloride sources of Cu, Zn, and Mn tended (*P =* 0.060) to have greater ADG over the 20-wk preweaning period compared with calves consuming sulfate sources of the same elements (1.09 vs. 1.06 kg/d; SEM = 0.013). Since supplement intake did not differ, this response suggests that the source of Cu, Zn, and Mn may have a biological impact beyond simply promoting greater intake. Spears et al. (2004) reported that Cu from tribasic Cu chloride (hydroxychloride Cu) had greater bioavailability than Cu sulfate in cattle.

Mineral status of cows and calves at the end of the 20-wk evaluation was not affected (*P ≥* 0.17) by source of Cu, Zn, and Mn (Table 6). Liver mineral concentrations were within the range of adequacy for both cows and calves (Puls, 1988). One notable exception may be liver Se concentration in calves (average = 0.61 mg/kg DM; SEM = 0.121). This concentration is marginally adequate by the threshold suggested by Puls (1988) and the Diagnostic Center for Population and Animal Health at Michigan State University (0.61 and 0.70 mg/kg DM, respectively). These marginal concentrations of liver Se are similar to those reported in our earlier studies (Arthington et al., 2014; Caramalac et al., 2017) and represent a meaningful and practical consideration for cow/calf production systems. When offered free choice, we expect cattle to consume salt-based mineral supplements in amounts that meet or exceed their Na requirement. In the current study, calf intake of free-choice mineral averaged 16.1 g/d resulting in 1.8 and 2.8 mg of Se intake daily for hydroxychloride and sulfate treatments, respectively (amounts differ due to differing Se concentration of test products; Table 2). From a Na selenite source, these amounts are insufficient to support Se adequacy in calves (Ranches et al., 2017). The outcome was different for cows. Because their voluntary intake was greater than calves, they experienced 7.7 and 10.7 mg of Se intake daily, which supported adequate Se status. Interestingly, this amount of supplemental Se intake exceeds the FDA maximum rate for beef cattle (3 mg of supplemental Se/d for free-choice mineral supplements; Code of Federal Regulations; Title 21, Vol. 6), so decreasing the amount of Se in the supplement, or taking measures to reduce intake is warranted. Unfortunately, these changes will only intensify the Se deficiency problem affecting the calves. Depending on the same free-choice mineral formulation to supply both cow and calf needs may be troublesome due to differences in voluntary intake, particularly when considering Se. This is concerning due to the prevalence of Se inadequacy in forages throughout the United States (Corah and Dargatz, 1996) and its essentiality for multiple physiological functions affecting cattle health and performance (Underwood and Suttle, 2001).

**Table 6.** Liver mineral concentrations of cows and calves provided free-choice, salt-based mineral supplements with hydroxychloride or sulfate sources of Cu, Zn, and Mn (Exp. 2)^1^

	Item	Hydroxychloride	Sulfate	SEM^2^	*P*-value
		mg/kg DM			
Cows	Co	0.31	0.30	0.044	0.85
	Cu	220	237	43.5	0.79
	Fe	477	383	42.8	0.17
	Mn	11.34	11.53	1.471	0.93
	Mo	3.06	3.33	0.370	0.62
	Se	1.30	1.07	0.176	0.40
	Zn	151	140	9.8	0.48
Calves	Co	0.20	0.20	0.041	0.95
	Cu	138	116	29.9	0.63
	Fe	382	335	55.1	0.57
	Mn	8.95	8.07	1.722	0.73
	Mo	2.25	2.25	0.487	1.00
	Se	0.60	0.61	0.121	0.97
	Zn	145	138	13.4	0.72

^1^Values are least square means. Liver biopsy samples were collected at the end of the study following 20 wk of supplementation.

^2^Largest SEM.

**Figure 3.**
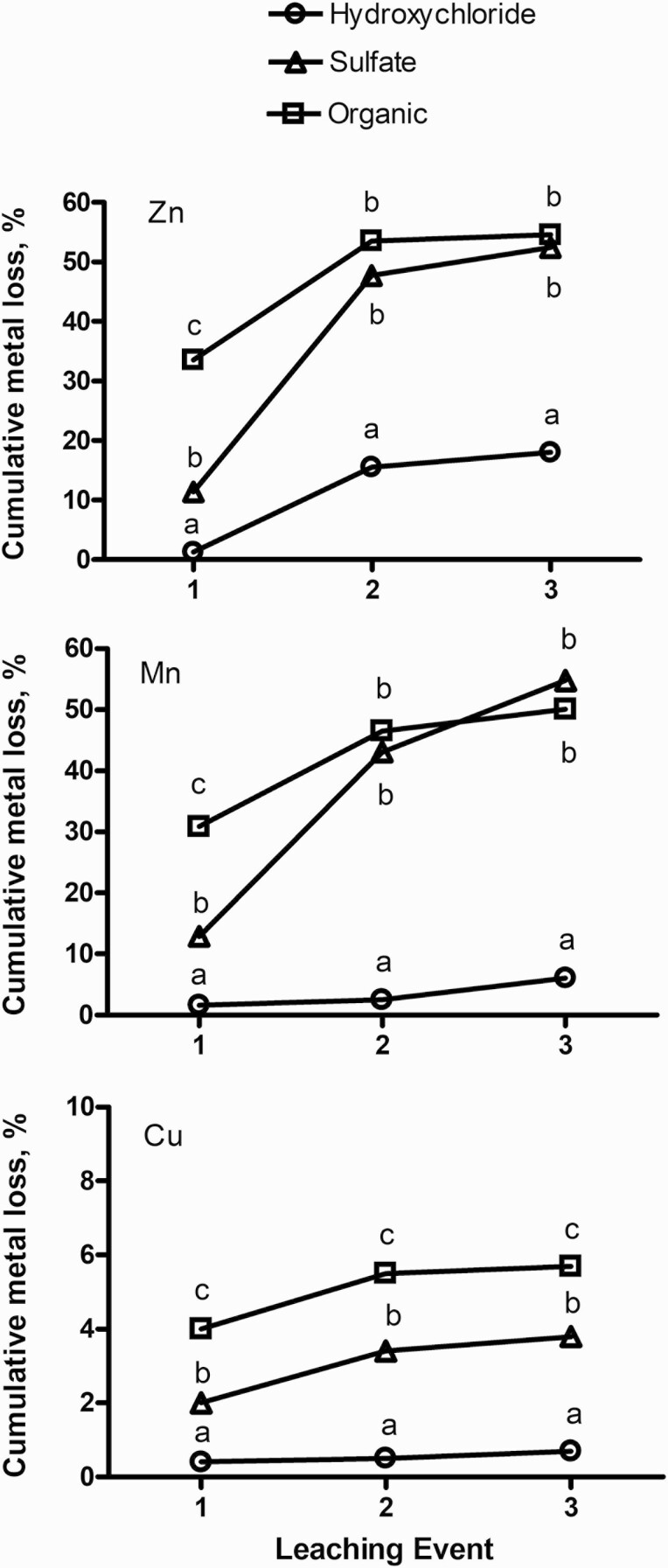
Cumulative metal loss from a complete, salt-based mineral supplement resulting from a 10.2-cm simulated precipitation event occurring over three separate 3.4-cm leaching events within a week. Metal source × leaching event; *P <* 0.001 for each of the three individual metals. Means with unlike superscripts within leaching event (i.e., day) and across mineral source differ (*P <* 0.05).

In Exp. 3, losses of Cu, Zn, and Mn were evaluated under controlled, rainfall-simulated conditions. Based on previous research comparing the solubility of Cu sulfate and Cu hydroxychloride (Spears et al., 2004), we sought to examine Cu, Zn, and Mn loss when complete, salt-based mineral formulations were exposed to a simulated rainfall of 10.2 cm. The total 10.2 cm of water was delivered in three equal events administered in 48-h intervals. Mineral loss is presented as a percent accumulative loss based on the amount present in the initial material (Figure 3). Overall, losses were greatest (*P <* 0.001) for Zn and Mn, compared with Cu, irrespective of source. For Cu, total cumulative loss was greatest for organic (5.8%), least for hydroxychloride (0.8%), with sulfate being intermediate (3.8%; Table 7). For Zn and Mn, total losses did not differ (*P ≥* 0.20) between sulfate and organic sources, but were 3.0 and 8.5× greater (*P <* 0.001), respectively, than hydroxychloride sources of these elements (Table 7). There was a treatment × time interaction (*P <* 0.05) for cumulative metal loss over the three precipitation simulation events. This interaction was the result of greater initial metal loss from the organic source of Cu, Zn, and Mn, compared with sulfate and hydroxychloride sources, in the first precipitation event (Figure 2). Solubility information regarding the organic mineral ingredient used in this study was not available. The American Association of Feed Control Officials (**AAFCO**) define the ingredient as a metal proteinate, which are described as products resulting from the chelation of a soluble salt with amino acids and/or partially hydrolyzed protein (AAFCO definition 57.23). Solubility may be impacted by the contribution of free amino acids as the addition of lysine and glycine (2:1 molar ratio of amino acid:Cu) greatly increases Cu solubility (Spears et al., 2004).

**Table 7.** Effect of Zn, Mn, and Cu source on cumulative metal loss (% of total) from complete, salt-based mineral supplement formulations exposed to a 10.2-cm simulated rainfall event

	Source of metal			
Metal	Sulfate	Organic	Hydroxychloride	Pooled SEM
Zn	52.3^a^	54.6^a^	18.0^b^	1.83
Mn	54.8^a^	50.1^a^	6.2^b^	1.45
Cu	3.8^a^	5.8^b^	0.8^c^	0.22

Three equal 3.4-cm rainfall simulations were applied oat 48-h intervals, totaling 10.2 cm.

^abc^Cumulative metal loss, means in a row with unlike superscripts differ; *P <* 0.05.

The results of these three studies illustrate that calves preferentially consume free-choice salt-based mineral supplements formulated with hydroxychloride sources of Cu, Zn, and Mn vs. sulfate or inorganic of the same elements, but only when provided a choice. Devoid of choice, which is practical under production conditions, free-choice intake is the same, but calf ADG is improved when consuming hydroxychloride vs. sulfate or organic sources of Cu, Zn, and Mn. Further research is warranted to elucidate the biological relevance of this calf BW gain response. Lastly, meaningful rainfall-induced changes in the nutrient composition of free-choice mineral supplements were revealed with losses considerably less for salt-based supplements formulated with hydroxychloride vs. sulfate our organic sources of Cu, Zn, and Mn. Consideration to ingredient source, weatherizing agents, and the use of feeding structures that protect the product from weather impacts or reduce losses of leached material are important to ensure the integrity of the product consumed.
